# Improving Communication for Older Adults During Disaster Events

**DOI:** 10.1093/geroni/igaa057.2425

**Published:** 2020-12-16

**Authors:** Allison Gibson, Nancy Kusmaul, Lisa Brown

## Abstract

Management of any disaster event (i.e., hurricanes, flooding, wildfires, viral pandemic) is a complicated task. An important and frequently overlooked aspect of efficient disaster response is effective information exchange among emergency managers, aging and social service providers, and those impacted by the disaster. Breakdowns in communication are regularly cited as failures in actual emergency situations. Older adults have unique challenges to effective communication during a disaster event. Further, messaging platforms may be inaccessible to older adults. Given the lack of data collected on the role of effective communication for older adults in a disaster event, we developed this symposium to highlight lessons learned for facilitating disaster communication among older community members and discuss how policy can further disaster communication efforts. The first presentation highlights the advantages of using targeted marketing communication channels to encourage older adults to prepare and recover from a disaster, as well as the value of collaborative partnerships in this communication. The second presentation introduces results from research collected from older adults on their use of social media to stay informed before and after Hurricane Matthew. The third presentation explores whether assisted living communities in Florida’s affected counties evacuated or sheltered in place in the context of emergency management communications concerning evacuation. The fourth presentation highlights the experiences of older persons in Houston’s communication post-Hurricane Harvey from an aging service provider’s perspective. The final presentation discusses disaster literacy and how this model can be used to enhance outcomes at all phases of a disaster. Disasters and Older Adults Interest Group Sponsored Symposium.

